# Location of a single histidine within peptide carriers increases mRNA delivery

**DOI:** 10.1002/jgm.3295

**Published:** 2020-12-21

**Authors:** Jiaxi He, Songhui Xu, Qixin Leng, A. James Mixson

**Affiliations:** ^1^ Department of Pathology, University Maryland School of Medicine University of Maryland Baltimore MD USA

**Keywords:** Non‐viral vector, peptide synthesis, RNA transfer, transfection

## Abstract

**Background:**

Previously, we determined that four‐branched histidine‐lysine (HK) peptides were effective carriers of plasmids and small interfering RNA. In the present study, we compared several branched HK carriers and, in particular, two closely‐related H3K4b and H3K(+H)4b peptides for their ability as carriers of mRNA. The H3K(+H)4b peptide differed from its parent analogue, H3K4b, by only a single histidine in each branch.

**Methods:**

A series of four‐branched HK peptides with varied sequences was synthesized on a solid‐phase peptide synthesizer. The ability of these peptides to carry mRNA expressing luciferase to MDA‐MB‐231 cells was investigated. With gel retardation and heparin displacement assays, the stability of HK polyplexes was examined. We determined the intracellular uptake of HK polyplexes by flow cytometry and fluorescence microscopy. The size and polydispersity index of the polyplexes in several media were measured by dynamic light scattering.

**Results:**

MDA‐MB‐231 cells transfected by H3K(+H)4b‐mRNA polyplexes expressed 10‐fold greater levels of luciferase than H3K4b polyplexes. With gel retardation and heparin displacement assays, the H3K(+H)4b polyplexes showed greater stability than H3K4b. Intracellular uptake and co‐localization of H3K(+H)4b polyplexes within acidic endosomes were also significantly increased compared to H3K4b. Similar to H3K(+H)4b, several HK analogues with an additional histidine in the second domain of their branches were effective carriers of mRNA. When combined with DOTAP liposomes, H3K(+H)4b was synergistic in delivery of mRNA.

**Conclusions:**

H3K(+H)4b was a more effective carrier of mRNA than H3K4b. Mechanistic studies suggest that H3K(+H)4b polyplexes were more stable than H3K4b polyplexes. Lipopolyplexes formed with H3K(+H)4b markedly increased mRNA transfection.

## Graphical abstract

Branched HK mRNA polyplexes with one additional histidine in a specific location show marked improvement in their cellular uptake, accumulation within endosomes, as well as their transfection efficiency. Those HK carriers lacking an extra histidine, which form mRNA polyplexes, have decreased stability, resulting in a low transfection.

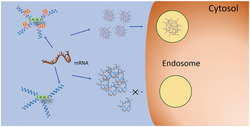

## 1 INTRODUCTION

Delivery of mRNA has been considered as an excellent alternative to plasmids because it can be translated to a protein in the cytosol without entering the nucleus to become functional. Therefore, mRNA can successfully express proteins in non‐dividing cells.^1^ Although degradability of mRNA may in some ways be advantageous to reduce toxicity,^2^, ^3^ the susceptibility of mRNA to enzymatic degradation with reduced translation accounts for significant problems. Consequently, the development of carriers that can protect mRNAs from degradation, facilitate cellular uptake and enhance buffering capacity to improve endosomal escape has a high priority. Among them, non‐viral carriers, including polymers and lipid‐based agents such as lipopolymers and liposomes, have been used for mRNA delivery.^4^, ^5^, ^6^, ^7^, ^8^ Of these, liposomes are the most studied and are effective carriers of mRNA.^1^, ^9^, ^10^, ^11^ For example, Zohra et al.^1^, ^11^ found that DOTAP liposomes coated with carbonate apatite showed high luciferase mRNA transfection efficiency in both mitotic and non‐mitotic cells.

There have been only a limited number of studies demonstrating the utility of polymers as mRNA carriers.^6^, ^7^, ^8^, ^12^, ^13^, ^14^, ^15^, ^16^, ^17^, ^18^, ^19^, ^20^, ^21^, ^22^, ^23^ Qiu et al.^21^ synthesized an RNA delivery vector, PEG12KL4, in which the synthetic cationic KL4 peptide was attached to a linear 12‐mer of PEG. With intratracheal administration, these carriers mediated significantly more effective mRNA transfection in the lungs of mice than naked mRNA. Moreover, based on the studies of Kataoka and colleagues,^12^ Chan et al.^22^ compared several repeating units of aminoethylene groups (2, 3, or 4) conjugated as side chains to a PEGylated polyaspartamide backbone. The carrier with the side branch of four‐repeating units, tetraethylenepentamine, had the best luciferase mRNA delivery efficiency *in vitro* and effectively delivered luciferase mRNA injected intracerebroventricularly with no significant immune response. Interestingly, by altering the alkyl length between amines, Jarzebinska et al.^6^ found an oligoalkylamine that significantly enhanced mRNA delivery. This oligoalkylamine had a high buffering capacity between pH 6.2 and 6.5, a pH range that has been associated with endosomal lysis and escape of nucleic acids. Several investigators have also utilized either peptide‐liposomes or lipopolymers to stabilize the vector to deliver mRNA *in vivo*. With few exceptions,^13^, ^15^ lipid‐polymer hybrids or liposome‐polymer combinations are required or at least greatly enhance the systemic delivery of mRNA.^6^, ^7^, ^8^, ^19^, ^20^, ^24^

Our laboratory has focused on developing histidine‐rich peptides to deliver nucleic acids, including mRNA. Although several groups have used histidine‐ or imidazole‐containing polymers to enhance the delivery of relatively small molecular weight small interfering RNA (siRNA) and large molecular weight plasmid DNA,^25^, ^26^, ^27^, ^28^, ^29^, ^30^, ^31^, ^32^ few of these modified polymers have been used to deliver mRNA. These studies have primarily focused on lipopolyplexes delivering mRNA for vaccines targeting tumors.^33^, ^34^, ^35^, ^36^ For example, Mockey et al.^35^ added histidylated liposomes to a PEGylated histidylated polylysine‐mRNA polyplex to form a lipopolyplex for melanoma mRNA delivery, which significantly enhanced the immune response to the translation product. Recently, this group decorated lipopolyplexes with a “tri‐antenna α‐d‐mannopyranoside” ligand, which increased their specificity toward dendritic cells and improved the antitumor immune response.^37^, ^38^

In the present study, we compared histidine‐rich peptide carriers and, in particular two close analogues, H3K4b and H3K(+H)4b peptides, for their ability as carriers of mRNA *in vitro*. H3K4b has four repeating motifs of ‐KHHH‐ in each branch, whereas the H3K(+H)4b has a similar repeating pattern but has an additional histidine in the second ‐HHHK motif of its branches (Table 1). We determined that the H3K(+H)4b peptide was a significantly better carrier of mRNA than H3K4b.

**TABLE 1:** Schematic structure and sequences of the four‐branched HK peptides

Four‐branched Polymers	Terminal sequence				Structure
H2K4b	**R = KHKHHKHHKHHKHHKHHKHK**				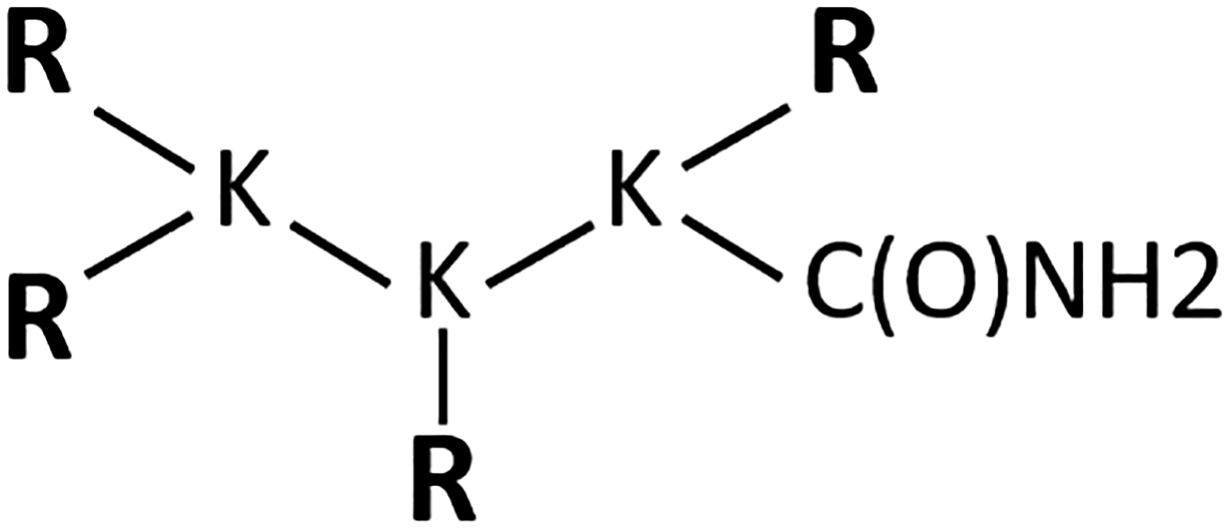
	4	3	2	1	
H3K4b	**R** = KHHHKHHHKHHHKHHHK				
H3K(+H)4b	**R** = KHHHKHHHKHHHHKHHHK				
H3k(+H)4b	**R** = kHHHkHHHkHHHHkHHHk				
H‐H3K(+H)4b	**R** = HKHHHKHHHKHHHHKHHHK				
HH‐H3K(+H)4b	**R** = HHKHHHKHHHKHHHHKHHHK				
H4K4b	**R** = KHHHHKHHHHKHHHHKHHHHK				
H3K(1+H)4b	**R** = KHHHKHHHKHHHKHHHHK				
H3K(3+H)4b	**R** = KHHHKHHHHKHHHKHHHK				
H3K(1,3+H)4b	**R** = KHHHKHHHHKHHHKHHHHK				

H and K represent l‐histidines and l‐lysines, respectively. The lower case “k” in the sequence of H3k(+H)4b represents d‐lysines. Extra histidines are underlined within the terminal branch sequences with H3K4b used as a reference. The numbers above the H3K4b peptide and analogues represent the four repeating motifs of the branch. Branched peptides emanate from a 3‐lysine core.

## 2 MATERIALS AND METHODS

### 2.1 Peptides

HK peptides were synthesized on a Ranin Voyager synthesizer (Tucson, AZ, USA) by the biopolymer core facility at the University of Maryland or by Genscript (Piscataway, NJ, USA) as described previously.^39^, ^40^ To ensure a purity of 90% or greater,^39^ peptides were analyzed by high‐performance liquid chromatography (Beckman Coulter, Fullerton, CA, USA) with System Gold operating software, using a Dynamax 21.4 X 250 mm C‐18 reversed‐phase preparative column (Varian, Palo Alto, CA, USA) with a binary solvent system. Further analyses of the peptides were carried out by ESI mass spectroscopy (LCMS‐2020; Shimadzu Corporation, Kyoto, Japan). The sequences of the HK peptides are shown in Table 1.

Nomenclature of four‐branched HK polymers: (i) for H3K4b, the dominant repeating sequence in its terminal branch is ‐HHHK‐, thus “H3K” is part of the name; the “4b” refers to the number of terminal branches; (ii) there are four ‐HHHK‐ motifs in each branch of H3K4b and analogues. The first motif is closest to the lysine core; (iii) H3K(+H)4b is a four‐branched analogue of H3K4b in which one extra histidine is inserted in the second ‐HHHK‐ motif of the terminal branch of H3K4b; (iv) H3K(1+H)4b and H3K(3+H)4b have an extra histidine in the first and third motifs, respectively; (v) for H3K(1,3+H)4b, there is an extra histidine in both the first and third motifs of the branches; (vi) for H‐H3K(+H)4b and HH‐H3K(+H)4b, these peptides are analogous to H3K(+H)4b, except they have one or two additional histidines at the N‐terminal ends of the branches; (vii) for H4K4b, the predominant pattern in the branches is ‐HHHHK‐; (viii) for H2K4b, the predominant pattern in the branches is –HHK‐.

### 2.2 In vitro mRNA transfection

Several HK peptides were examined for their ability to carry a luciferase‐expressing mRNA (CleanCap Firefly Luciferase mRNA; Trilink Biotechnologies, Inc., San Diego, CA, USA) into MDA‐MB‐231 cells (America Type Tissue Culture, Manassas, VA, USA). In brief, 3 × 10^4^ cells were plated into a 24‐well plate containing 500 μl of Dulbecco’s modified Eagle’s medium (DMEM) and 10% fetal bovine serum (Thermo Fisher Scientific, Waltham, MA, USA). After 24 hours, when the cells were 60–80% confluent, the media in each well was changed to Opti‐MEM (Thermo Fisher Scientific). To prepare HK polyplexes, mRNA (1 μg) in 50 μl of Opti‐MEM was briefly mixed well with one of the HK peptides (4–12 μg) and maintained at room temperature for 30 minutes. This polyplex was then added dropwise to the cells. After 4 hours, the Opti‐MEM media was removed and replaced with 0.5 ml of DMEM/10% serum. Twenty‐four hours later, the cells were lysed, and luciferase activity (Promega Corporation, Madison, WI, USA) was measured.^28^ Also, a negative control (mRNA without the carrier) was added to the cells and luciferase activity measured 24 hours later.

Transfection with HK lipopolyplexes was carried out in a manner similar to that described above, with a few exceptions. In brief, the HK peptide (4 μg) in Opti‐MEM was mixed initially with mRNA (1 μg) for 30 minutes. This was followed by adding the DOTAP cationic liposome (1,2‐dioleoyl‐3‐trimethyl ammonium‐propane; 1 μg; Roche, Basel, Switzerland) for an additional 30 minutes. The Opti‐MEM mixture (50 μl) was then added dropwise to the cells.

### 2.3 Acid–base titration

After the polymer solution [H2K4b, H3K4b, H3K(+H)4b and H4K4b] was adjusted to pH 3.0 (5 mg/ml; initial volume 1 ml), aliquots (5 μl) of NaOH (0.05 m) were stepwise added with the pH measured (FiveEasy™ meter; InLab Solids Pro‐ISM pH electrode; Mettler Toledo, Columbus, OH, USA). Titration was stopped at approximately pH 9.0.

### 2.4 Cell viability assay

MDA‐MB‐231 cells were seeded at 5.0 × 10^4^ per well in a 24‐tissue culture plate and incubated overnight in DMEM supplemented with 10% fetal bovine serum (FBS) serum. The media was then changed to Opti‐MEM and the cells were treated with either the polymer (4 μg) or the polyplex (4 μg of HK; 1 μg of mRNA) for 5 hours. After the media was changed to DMEM/10% FBS for 19 hours, the cell viability was measured using the trypan blue cell exclusion assay (Trypan Blue solution, 0.4%; Sigma‐Aldrich, St Louis, MO, USA).^41^

### 2.5 Gel retardation assay

Various amounts of HK peptides were mixed with 1 μg of mRNA and incubated for 30 minutes at room temperature. Specifically, the following HK/mRNA ratios (w/w) were prepared in water: 1/2; 1/1; 2/1, 4/1 and 8/1. After 30 minutes, the HK polyplex was loaded onto the gel (20 μl; 1% agarose, Sigma‐Aldrich; 10X BlueJuice Gel loading buffer, Thermo Fisher Scientific), and electrophoresis was then carried out at a constant voltage of 75 V for 30 min in Tris‐acetate‐ethylenediaminetetraacetic acid (TAE) buffer (Quality Biologicals, Gaithersburg, MD, USA). The mRNA was stained with Sybr Gold Nucleic Acid dye (SG, 1X) (Thermo Fisher Scientific) for 30 minutes before exposure to the ultraviolet imager (ChemiDoc Touch; Bio‐Rad, Hercules, CA, USA).

### 2.6 Heparin displacement assays

Heparin displacement assays of mRNA polyplexes were performed with the dye intercalation assay and gel electrophoresis. A fluorescence assay assessed polyplexes of HK and mRNA (4:1 w/w ratio; polymer:mRNA) formed in RNAse/DNAase free water (Corning, Manassas, VA, USA). Polyplexes were prepared as described previously, followed by the addition of diluted Syber Gold. For detection, working dilutions of the polyplexes (1/5 of volume), water (3/5) and Sybr Gold dye (1/5, 0.2X) were incubated for 5 minutes, and fluorescence was measured by a fluorimeter (excitation = 497 nm, emission = 520 nm) (Synergy Mx; BioTek, Winooski, VT, USA). The control sample was prepared with the same amount of mRNA, water and Sybr Gold dye. For the heparin displacement, instead of water, heparin salt (Sigma‐Aldrich) solutions at different concentrations (0.5, 1, 1.5, 2 and 3 μg/μl) were used, and the polyplexes were incubated at 37°C for 30 minutes before the addition of Sybr Gold.

Displacement of mRNA from polyplexes with heparin was also performed with gel electrophoresis. After polyplexes were formed, different concentrations of heparin (0.5, 1.0, 1.5, 2.0 and 3.0 μg/μl; volume 20 μl) were incubated with these at 37°C for 30 min. The polyplexes were then loaded on the agarose gel (1% gel; 20 μl loading volume; 10X BlueJuice Gel loading buffer) and electrophoresis was carried out followed by staining with SG as described above. Images were acquired via an ultraviolet imager (ChemiDoc Touch; Bio‐Rad).

### 2.7 In vitro uptake of HK polyplexes by fluorescence microscopy

With the mRNA labeled with Cy5, HK polyplexes at a ratio of 4:1 (HK:mRNA) were prepared as described for *in vitro* transfection. The labeled polyplexes were incubated with MDA‐MB‐231 cells for four hours in Opti‐MEM. After the cells were washed with phosphate‐buffered saline (PBS) (Quality Biologicals, Gaithersburg, MD, USA), they were incubated for 30 minutes with LysoTracker Green DND‐26 (Cell Signaling Technology, Inc., Danvers, MA, USA), a dye that stains acidic endosomes and lysosomes. Then after the cells were washed twice with PBS and once with 1% Triton‐X, they were fixed (4% formalin/1% glutaraldehyde) and the nuclei were stained with chromatin dye Hoechst 33342 (Invitrogen, Carlsbad, CA, USA). Images were obtained with a Nikon TE2000‐S (Nikon, Tokyo, Japan) with a mercury lamp light source usng the filter sets: Ex‐357(20)/Em‐460(60) (Hoechst); Ex‐480(30)/Em‐535(45) (Lysotracker green DND‐26); Ex‐620(50)/Em‐690(50) (Cy5‐labeled‐mRNA). Red/green ratios were measured on 20 intracellular acidic vesicles (one per cell) using ImageJ, version 1.52.^42^

### 2.8 In vitro uptake of HK polyplexes by flow cytometry

Intracellular uptake of polymer–mRNA complex in MDA‐MB‐231 was measured by flow cytometry. Twenty‐four hours before the treatment, cells were plated in a 24‐well plate. The polymer‐mRNA polyplex was formed in Opti‐MEM at a ratio of 4:1 at room temperature for 30 minutes. After the cell culture medium was replaced with DMEM/10% FBS, H3K(+H)4b‐ or H3K4b‐mRNA polyplexes (cyanine 5'‐labeled mRNA; Trilink Biotechnologies, Inc.) were added to the cells. At several time points (1, 2 and 4 hours), transfected cells were harvested, fixed with 4% formalin/1% glutaraldehyde, and resuspended in PBS buffer for analysis. The results from the fluorescently labeled MDA‐MB‐231 cells were then acquired using Cytoflex (Beckman Coulter) and analyzed using CytExpert, version 2.3.0.84 (Beckman Coulter) on the flow cytometer.

### 2.9 Stability of HK polyplexes to enzymatic degradation

After preparation of the H3K4b‐ or H3k(+H)4b‐mRNA polyplexes [w:w; HK (0.5, 1 or 4 μg):mRNA (1 μg)], these polyplexes were incubated with trypsin (0.025%) for 30 or 60 minutes. The HK polyplexes were then loaded on a 1% agarose gel and electrophoresis was carried out at 75 V for 30 minutes in TAE buffer. The gel was stained in a TAE buffer containing ethidium bromide (1 μg/ml) for 10 minutes.

### 2.10 Particle size, polydispersity index (PDI) and zeta potential

The size, PDI and zeta potential were determined with the Zetasizer (Malvern, Westborough, MA, USA) and analyzed with Zetasizer, version 6.2 (Malvern). Using dynamic light scattering at a 90° angle, the size of the particles were reported as the z‐average diameter from the intensity‐weighted size distribution. Prior to the measurements, the samples were equilibrated to 25°C for 2 minutes. Each measurement had at least 10 sub‐runs under the automatic mode of the software. The particle size, PDI and zeta potential data point represent the mean ± SD of three measurements. After mixing HK peptides (4 μg) and mRNA (1 μg) in 100 μl of defined media (Opti‐MEM, water, or DMEM/8% FBS) for 30 minutes, 100 μl of additional defined media was added to the polyplex solution (total volume 200 μl) to measure the size and PDI. To determine the zeta potential, 800 μl more of the media was added (total volume 1000 μl), mixed gently and then added to the disposable zeta cell.

### 2.11 Statistical analysis

The results, reported as the mean ± SD, represent three separate data measurements unless otherwise indicated. Except where stated, results were analyzed using a two‐tailed *t*‐test. *p* < 0.05 was considered statistically significant (SigmaPlot, San Jose, CA, USA).

## 3 RESULTS

### 3.1 H3K(+H)4b is a significantly better carrier than H3K4b

**FIGURE 1:**
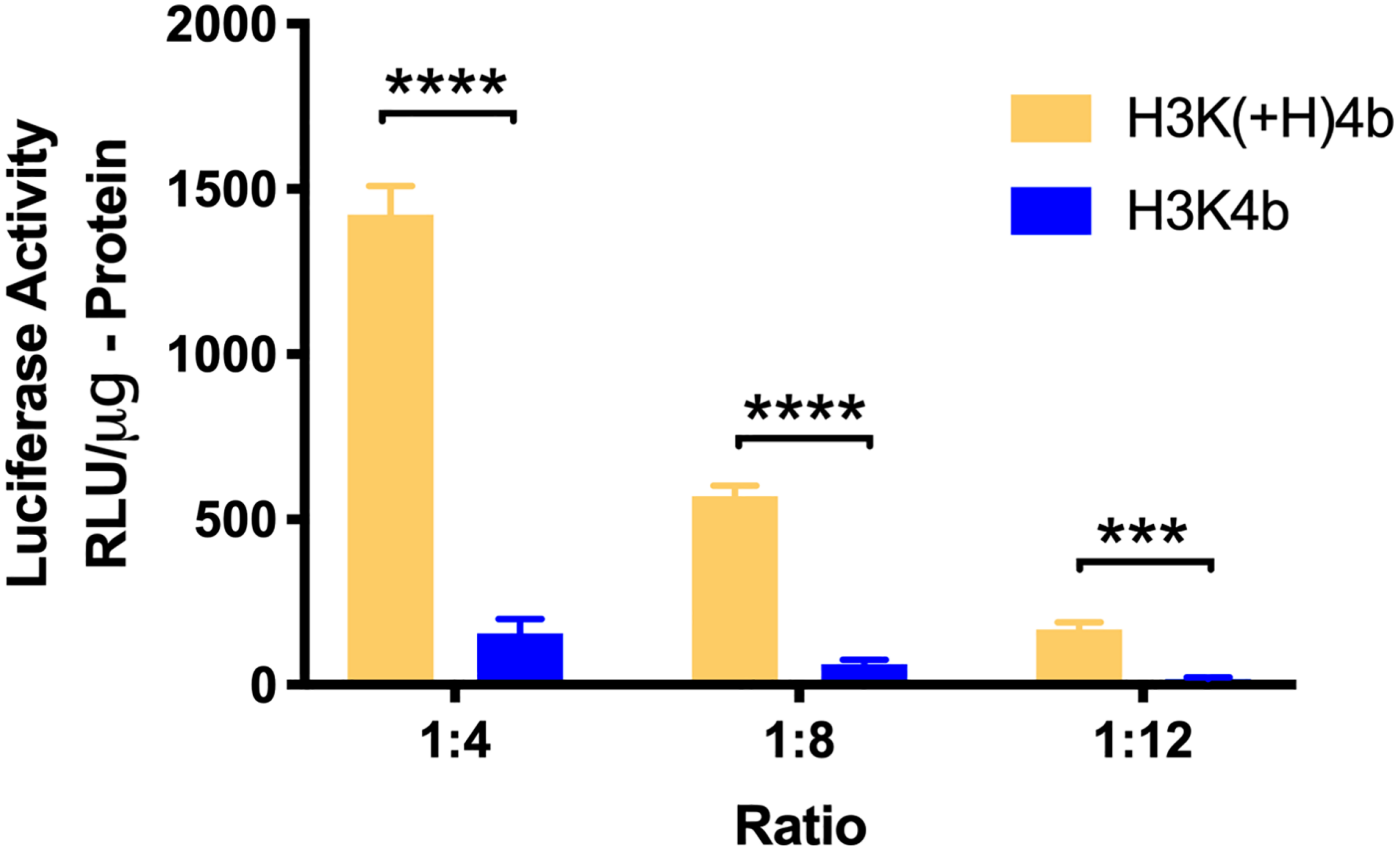
Comparison of H3K(+H)4b and H3K4b peptides as carriers of mRNA. To form polyplexes, the mRNA (1 μg) was mixed with three different ratios of the HK (4, 8 and 12 μg) polymer for 30 minutes. The mRNA polyplexes were then added to the cells as described in the Materials and methods, and luciferase activity was measured 24 hours later. ***p < 0.001; ****p < 0.0001, H3K(+H)4b versus H3K4b

**FIGURE 2:**
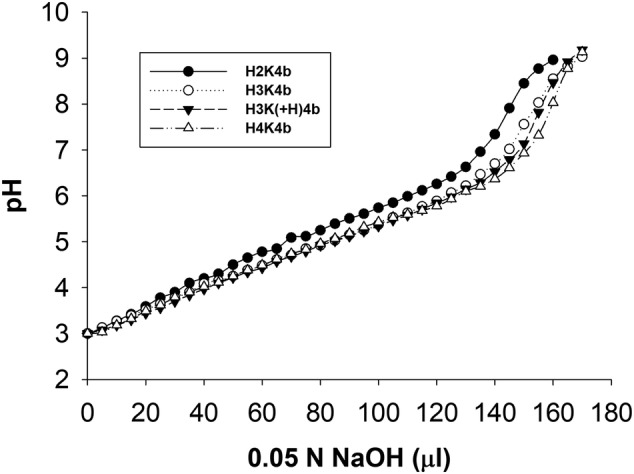
Titration of different HK peptide solutions. Solutions of polymers (5 mg/ml) [H2K4b, H3K4b, H3K(+H)4b and H4K4b] were adjusted to pH 3 (initial volume 1 ml) and then 5‐μl aliquots of 0.05 N NaOH were stepwise added and the pH was measured

Both H3K4b and H3K(+H)4b have shown promise as carriers of nucleic acids *in vitro*.^40^, ^43^ Despite these previous findings, H3K(+H)4b was markedly better as a carrier of mRNA compared to its close H3K4b analogue (Figure 1). At the ratio of 4:1 (HK:mRNA; w:w), luciferase expression was 10‐fold greater with the H3K(+H)4b than with the H3K4b peptide in MDA‐MB‐231 cells. At these HK mRNA ratios, neither polyplex showed cytotoxicity toward MDA‐MB‐231 cells (see Supporting information, Table S1). Moreover, the buffering capacity does not appear to be an essential factor in their transfection differences because the percentage of histidines (by weight) in H3K4b and H3K(+H)4b is 68.9% and 70.6%, respectively. Furthermore, the pH titration curves of H3K4b and H3K(+H)4b corroborated minimal differences in their buffering profile (Figure 2).

### 3.2 Gel retardation and heparin displacement assays indicate differences in their stability

**FIGURE 3:**
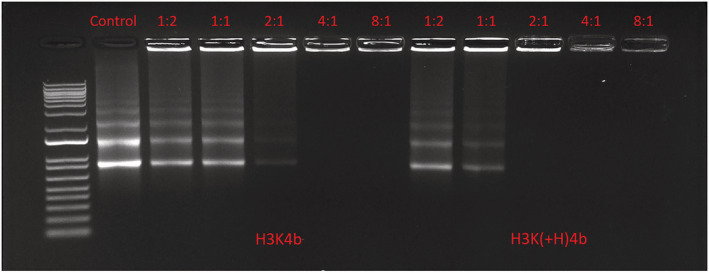
Gel retardation assay. After H3K(+H)4b or H3K4b carriers were mixed with mRNA at the various ratios of mRNA (w:w; peptide:mRNA, 1 μg) for 30 minutes, the polyplexes were loaded onto the gel (1% agarose). Electrophoresis was carried out at a constant voltage of 75 V for 30 minutes in TAE buffer and then stained with Sybr gold as described in the Materials and methods

The retardation assay showed the effect of different weight ratios of mRNA and HK polypeptides (Figure 3). The retardation effect increased with higher HK peptide to mRNA weight ratios. Free mRNA and partially retarded mRNA was markedly less at the ratios of 1:2 and 1: 1 (w:w; peptide:mRNA) of H3K(+H)4b compared to the same ratios of H3K4b. With ratios of 2:1 and 4:1, the mRNA was completely entrapped by the H3K(+H)4b polyplex, whereas with the ratio of 4:1, the mRNA was completely retarded by the H3K4b polyplex. These results suggest that the H3K(+H)4b forms a more stable polyplex, and this may play a role with respect to why H3K(+H)4b is more effective as a carrier compared to H3K4b.

**FIGURE 4:**
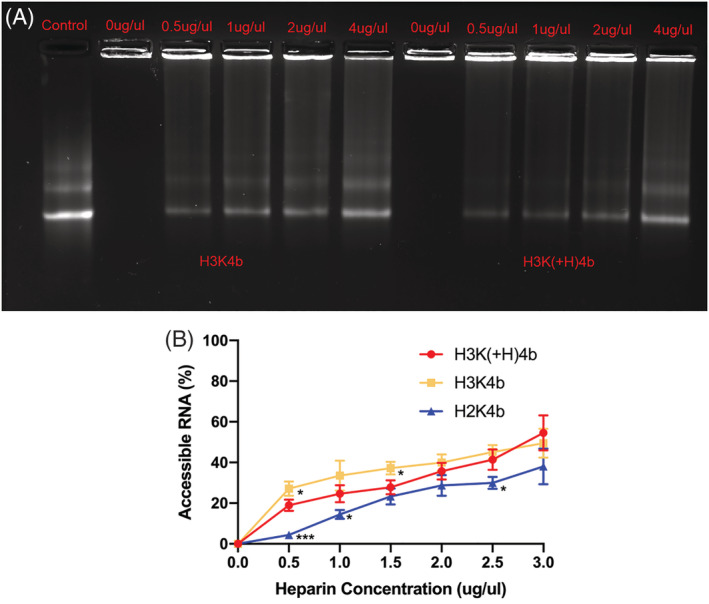
Heparin displacement assays. After polyplexes were formed (w:w; HK:mRNA; 4 μg:1 μg), different concentrations of heparin (0, 0.5, 1, 2 and 4 μg/ml) were incubated with these for 30 minutes. (A) Gel retardation. The polyplexes were loaded on the agarose gel (1%), and electrophoresis was carried out and stained with Sybr gold. (B) Fluorescent dye intercalation. After the polyplexes were formed and incubated with several concentrations of heparin, the Sybr gold nucleic acid dye was incubated with the polyplexes for 5 minutes. Fluorescence was then measured by a microplate fluorimeter (λ_excitation_ = 497 nm, λ_emission_ = 520 nm) (Synergy Mx; BioTek). *p < 0.05; ***p < 0.001; H3K4b or H2K versus H3K(+H)4b

Further confirmation that the H3K(+H)4b peptide binds more tightly to the mRNA was demonstrated with the heparin‐binding assay (Figure 4A, 4B). Particularly at the lower concentrations of heparin, mRNA was released by the H3K4b more rapidly than by the H3K(+H)4b peptide. These data, together with the size of polyplexes in different media (Table 2), suggest that H3K(+H)4b polyplexes may be more stable than the H3K4b polyplexes. Nevertheless, if a peptide such as H2K4b forms a polyplex that is too stable, this may also reduce mRNA transfection (Figure 4B). Release of the HK polymers, including H2K4b, from the polyplex is likely critical for the interaction of the HK polymer with endosomal membrane with subsequent lysis of the endosomes.^36^ Interestingly, other HK polyplexes that showed effective mRNA transfection had stabilities similar to the H3K(+H)4b polyplexes when exposed to heparin (see Supporting information, Figure S1). These HK carriers [H‐H3K(+H)4b and HH‐H3K(+H)4b] with a transfection similar to H3K(+H)4b had an additional histidine in their second domain. Moreover, the H3K(3+H)4b polyplex, which was an ineffective carrier of mRNA, had a stability similar to the H3K4b polyplex. Because we observed different stabilities between the H3K4b and H3K(+H)4b polyplexes, we investigated whether the sizes of these polyplexes varied based on the media in which they were prepared. Both H3K4b and H3K(+H)4b polyplexes had a similar size and PDI in water, although, when they were prepared in media with higher salt and/or serum (8%), H3K4b polyplexes were markedly larger (Table 2).

**TABLE 2:** The size, zeta potential and PDI of the polyplexes in different media

	Opti‐MEM	Water	DMEM/8%FBS
**Size (nm)**			
H3K(+H)4b	1,004 ± 61.1	234.9 ± 2.7	289 ± 38.2
H3K4b	2,030.7 ± 117.3	199.9 ± 0.8	578 ± 80.5
**Zeta potential (mV)**			
H3K(+H)4b	2.92 ± 1.83	20.77 ± 1.12	−12.7 ± 1.04
H3K4b	−4.20 ± 2.78	16.47 ± 0.74	−12.1 ± 2.22
**PDI**			
H3K(+H)4b	0.362	0.212	0.307
H3K4b	0.466	0.171	0.421

H3K(+H)4b and H3K4b peptides (4 μg) in complex with mRNA (1 μg) were mixed with either Opti‐MEM, water or DMEM/8% FBS (100 μl). After 1 hour, the size, PDI, and zeta potential were measured. With DMEM/8% FBS, endogenous lipid particles were not found and, consequently, this medium did not interfere with the size measurements of HK polyplexes.

### 3.3 Increased intracellular localization of H3K(+H)4b polyplexes compared to H3K4b

**FIGURE 5:**
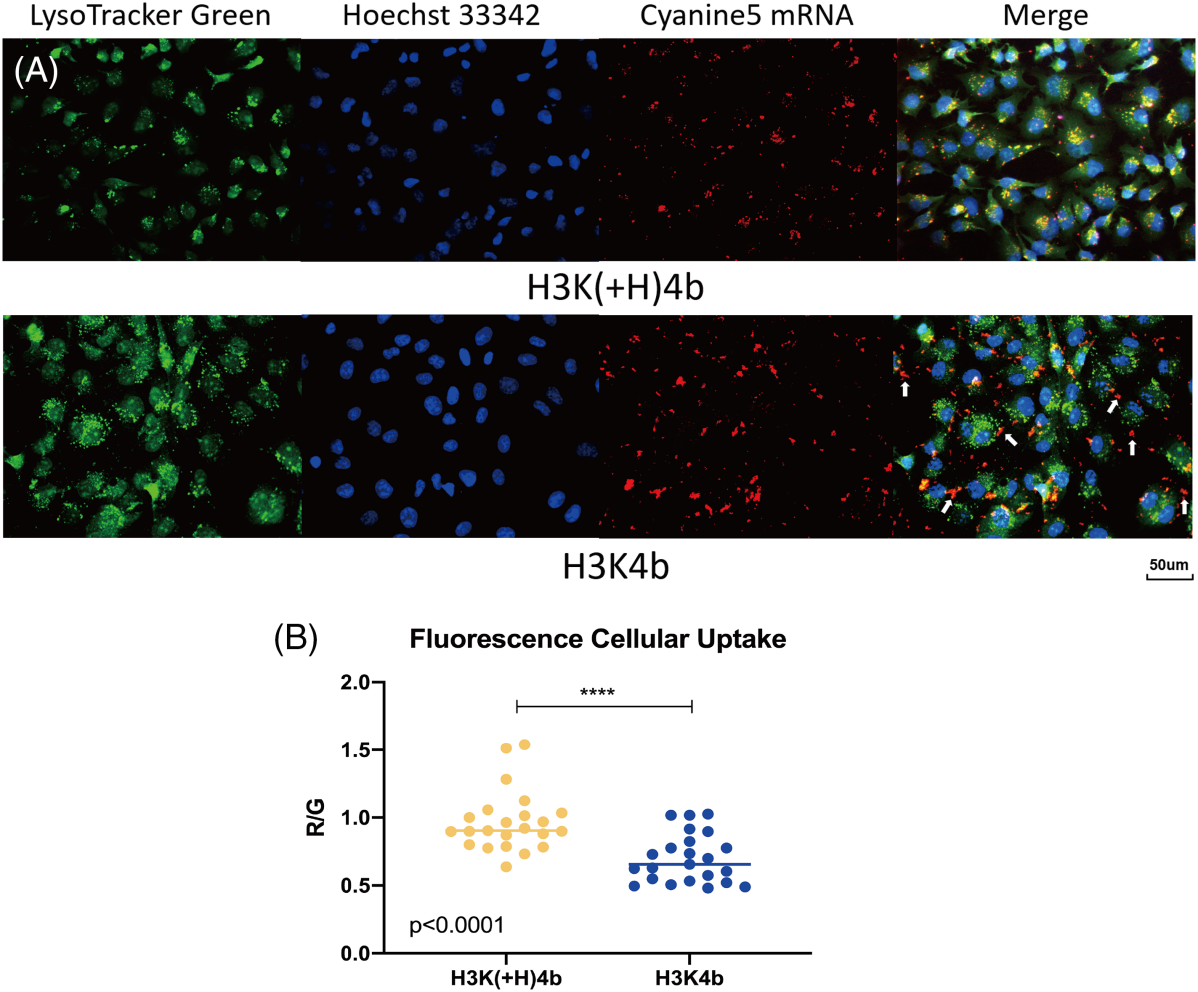
Fluorescence images of different mRNA polyplexes within acidic vesicles of MDA‐MB‐231 cells. (A) Four hours after transfection with labeled mRNA by H3K(+H)4b (upper) or H3K4b (lower) carriers, intracellular acidic vesicles were stained with Lysotracker green. The cells were then fixed, and the nuclei were stained with Hoesch 33342 dye. Images were obtained with a Nikon TE2000‐S using a mercury lamp light source and the filter sets delineated in the Materials and methods (green, acidic vesicles; red, cynanine 5‐labeled mRNA; blue, nuclei labeled with Hoescht dye). Lysotracker green, which accumulates within acidic endocytic vesicles, showed significantly greater overlap with cyanine 5‐labeled mRNA in which H3K(+H)4b was the carrier. Arrows indicate the irregularly shaped mRNA aggregates frequently observed with H3K4b carrier. (B) Analysis of the amount of HK (H3K4b or H3K(+H)4b) polyplexes within acidic endosomal vesicles of MDA‐MB‐231 cells. Images of HK polyplexes labeled with cyanine5 (red emission) were imported into endosomal vesicles (green emission). The red/green ratios were measured on 20 intracellular acidic vesicles using ImageJ. The uptake of H3K(+H)4b polyplexes into acidic vesicles were significantly more than H3K4b polyplexes. Mann–Whitney rank sum test: *p* < 0.001, H3K(+H)4b versus H3K4b polyplexes

With the mRNA labeled with cyanine‐5, we compared the uptake of H3K4b and H3K(+H)4b polyplexes into MDA‐MB‐231 cells using flow cytometry. At different time points (1, 2 and 4 hours), the H3K(+H)4b polyplexes were imported into the cells more than H3K4b polyplexes (see Supporting information, Figure S2). Similar to these results, fluorescence microscopy indicated that H3K(+H)4b polyplexes localized within the acidic endosomal vesicles significantly more than H3K4b polyplexes [H3K4b versus H3K(+H)4b; *p* < 0.001] (Figure 5). Interestingly, irregularly‐shaped H3K4b polyplexes, which did not overlap endocytic vesicles, were likely extracellular aggregates and were not observed with H3K(+H)4b polyplexes (Figure 5A). As evidenced by the heparin displacement assay (Figure 4; see also Supporting information, Figure S1), the reduced stability of H3K4b‐mRNA polyplexes provides a rationale for their irregular‐shapes compared to the H3K(+H)4b polyplexes.

### 3.4 Transfection of mRNA with HK carriers with extra histidine in the second motif is essential for mRNA transfection

**FIGURE 6:**
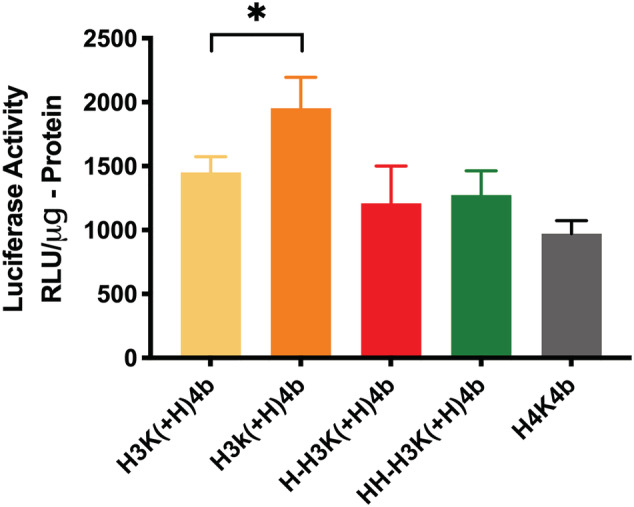
Comparing H3K(+H)4b and other four‐branched HK peptides for mRNA transfection. After polyplexes were formed with HK peptides (4 μg) and luciferase‐expressing mRNA (1 μg) and added to cells, luciferase activity was measured 14 hours later. These HK peptides contained an additional histidine in the second motif. H3k(+H)4b was the most effective peptide carrier of mRNA (H3k(+H)4b versus H3K(+4b). *p < 0.05

All the HK peptides with an extra histidine in the second ‐HHHK motif of the branches were effective carriers of mRNA (Figure 6; see also Supporting information, Table S2). Of these peptides, H3k(+H)4b was determined to be the optimal carrier of mRNA [H3k(+H)4b versus H3K(+H)4b; *p* < 0.05]. With this peptide, the l‐lysines were replaced with d‐lysines, and the enhanced stability of this polyplex may be the reason why this peptide was a better carrier than H3K(+H)4b. This was based on prior antimicrobial studies in which replacement of l‐lysines with d‐lysines suggested that H3k4b [a close analog of H3k(+H)4b] was more stable to enzymatic degradation, had greater antimicrobial activity, and had no observed cytotoxicity to human cells.^44^ Exposure to trypsin provided further support that the H3k(+H)4b‐mRNA polyplexes had enhanced stability to enzymatic degradations compared to the H3K(+H)4b polyplexes (see Supporting information, Figure S3).

Interestingly, additional histidines in locations other than the second motif do not appear to be a critical factor in enhancing mRNA transfection (Figure 6; see also Supporting information, Table S2). For example, H4K4b with twelve more histidines per peptide than H3K(+H)4b did not enhance mRNA transfection. Notably, the percentage of histidine content in H3K(+H)4b and H4K4b peptides was approximately 70.5% and 75%, respectively, and their similar buffering capacity was corroborated with the pH titration profile (Figure 2). Moreover, H‐H3K(+H)4b and HH‐H3K(+H)4b peptides with additional histidines did not improve mRNA transfection more than H3K(+H)4b. Nevertheless, these three peptides [H4K4b, H‐H3K(+H)4b and HH‐H3K(+H)4b] with a histidine in the second domain were effective carriers of mRNA similar to the H3K(+H)4b carrier.

**FIGURE 7:**
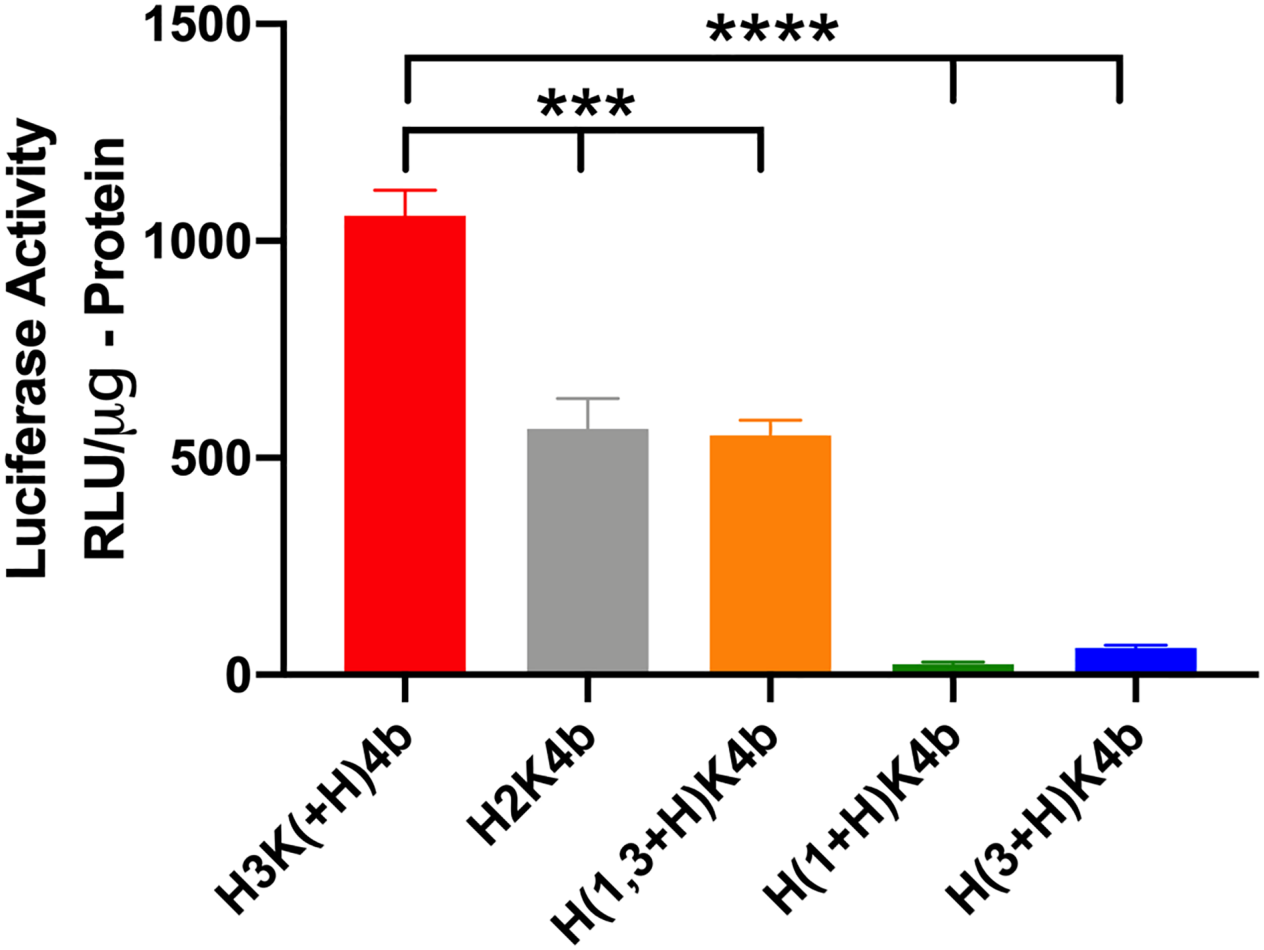
Comparison of H3K(+H)4b with branched peptides without an additional histidine in the second motif. Polyplexes were formed with mRNA (1 μg) and various HK peptides (4 μg) and, after their addition to cells, luciferase activity was determined 24 h later. H3K(1+H)4b and H3K(3+H)4b peptides have an extra histidine in the first and third domains, respectively. H3K(1,3+H)4b has two additional histidines: one in the first and the other third motif. Notably, the peptides [H3K(1+H )4b, H3K(3+H)4b and H3K(1,3+H)4b] do not have an extra histidine in the second motif. Unlike H3K(+H)4b, the predominant repeating motif of H2K4b in its branch is ‐HHK. ***p < 0.001, H3K(+H)4b versus H2K4b or H3K(1,3+H)4b; ****p < 0.0001, H3K(+H)4b versus H3K(1+H)4b or H3K(3+H)4b motifs

When the branched HK peptides with predominant pattern of ‐HHK‐ did not have an additional histidine in the second domain, mRNA transfection was markedly reduced (Figure 7; see also Supporting information, Tables 1 and S2). For example, although the H3K(1,3+H)4b peptide has an additional histidine in its first and third motif (compared to H3K4b) (Table 1), it does not have an extra histidine in the second motif. H3K(1,3+H)4b was approximately 2.5‐fold less effective in transfecting mRNA compared to H3K(+H)4b (*p* < 0.001). Moreover, although H3K(1,3+H)4b and H‐H3K(+H)4b had the same number of histidines and lysines per branch, H‐H3K(+H)4b was markedly more effective as a carrier of mRNA (Figures 6, 7). By contrast to the H3K(1,3+H)4b peptide, the H‐H3K(+H)4b peptide has an extra histidine in the second domain.

Similar to H3K4b and H3K(1,3+H)4b peptides, two other peptide carriers [H3K(1+H)4b and H3K(3+H)4b] that did not have an additional histidine in the second domain were poor carriers of mRNA (Figure 7 and Tables 1; see also Supporting information, Table S2). H3K(1+H)4b and H3K(3+H )4b have an extra histidine in first and third domains, respectively. Thus, the location of the histidines in the branches appears to be important.

Although the data for Figures 6 and 7 were obtained with the ratio of 4:1 (w/w, HK:mRNA), analogous results were generally found at 8:1 and 12: 1 ratios during the initial screening (see Suppoting information, Table S2). An exception was with H2K4b, a branched peptide with a predominant sequence of ‐HHK. Although the H2K4b carrier resulted in a similar, yet low, transfection of mRNA compared to H3K(1,3+H)4b at the ratio of 4:1 (Figure 7), transfection with H2K4b at the ratios of 8:1 and 12:1 was further reduced (see Suppoting information, Table S2). Compared to other branched HK polymers, H2K4b had the highest percentage of lysines.

**FIGURE 8:**
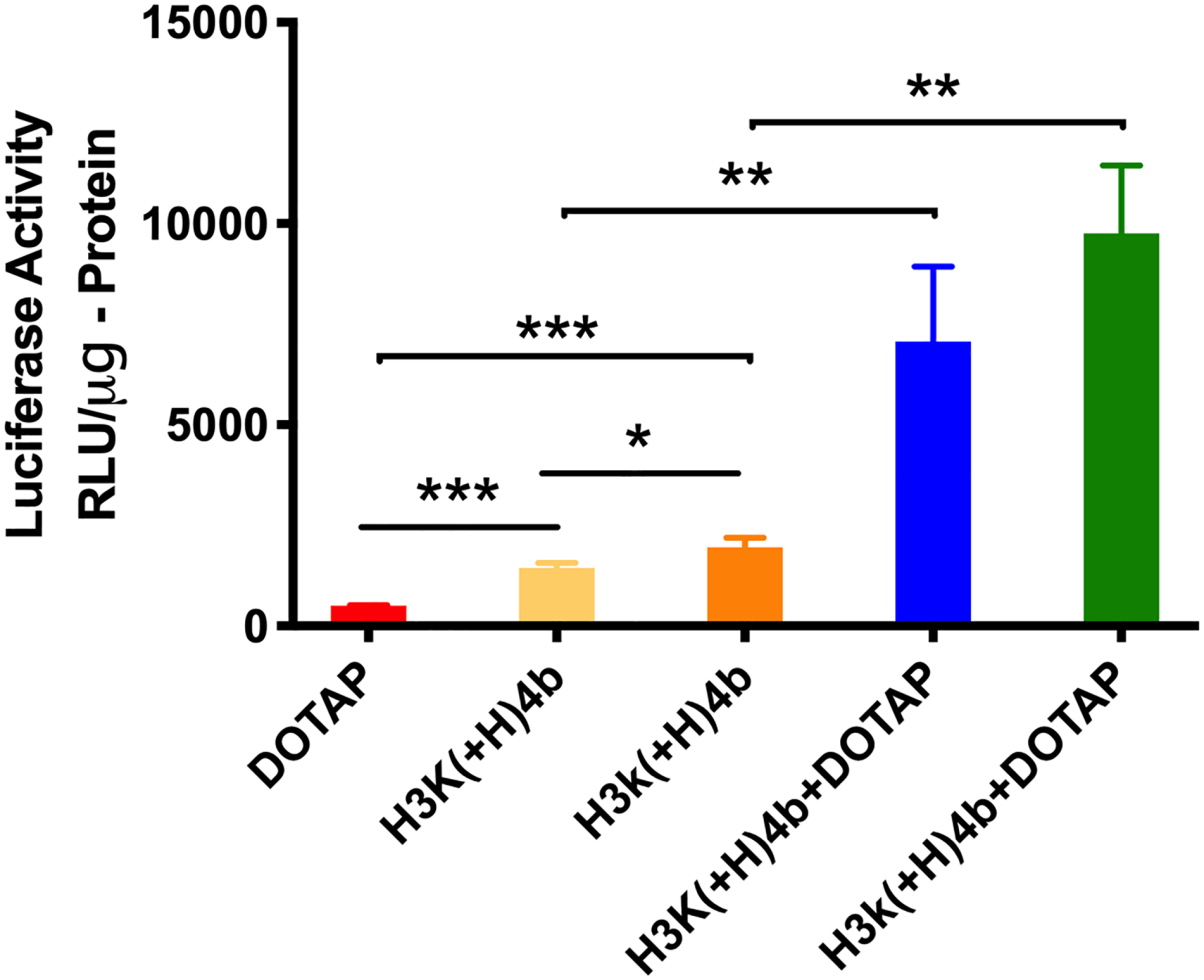
Comparison of mRNA transfection with DOTAP and several HK peptides. To form lipopolyplexes, the HK peptides (4 μg) in Opti‐MEM were initially mixed with mRNA (1 μg) for 30 minutes. DOTAP liposomes (1 μg) were then added, gently mixed and allowed to stand for an additional 30 min. Polyplexes were prepared as described previously. These complexes in Opti‐MEM (50 μl) were then added to the cells and, 24 hours later, the luciferase activity was measured. *p < 0.05; **p < 0.01; ***p < 0.001

Previously, we determined that HK peptides and cationic liposomes (i.e. DOTAP) significantly increased transfection with plasmids^45^ and, consequently, we investigated whether these liposomes together with HK peptides enhanced mRNA transfection. Notably, the H3K(+H)4b and H3k(+H)4b carriers were significantly better carriers of mRNA than the DOTAP liposomes (*p* < 0.001) (Figure 8; see also Supporting information, Figure S4). We determined that the combination of H3K(+H)4b and DOTAP liposomes was synergistic in the ability to carry mRNA into MDA‐MB‐231 cells (Figure 8; see also Suppoting information, Figure S4). The combination was approximately 3‐fold and 8‐fold more effective as carriers of mRNA than the polymer alone and the liposome carrier, respectively [H3K(+H)4b/liposomes versus liposomes or H3K(+H)4b; *p* < 0.01]. Notably, not all HK peptides demonstrated improved activity with DOTAP liposomes. The combination of H3K4b and DOTAP carriers was less effective than the DOTAP liposomes as carriers of luciferase mRNA (see Supporting information, Figure S4).

As stated previously, the d‐isomer, H3k(+H)4b, was the most effective polymeric carrier (Figure 6). The d‐isomer/liposome carrier of mRNA was almost 4‐fold and 10‐fold more effective than the H3k(+H)4b alone and liposome carrier, respectively (Figure 8)**.** Although the d‐H3k(+H)4b/liposome combination was modestly more effective than the l‐H3K(+H)4b/liposome combination, this comparison was not statistically different.

## 4 DISCUSSION

Diverse carriers are usually required for different nucleic acids.^46^, ^47^, ^48^, ^49^, ^50^, ^51^ For example, the large molecular weight branched polyethylenimine (PEI) (25 kDa) is an excellent carrier for plasmid DNA but not for mRNA. By decreasing the molecular weight of PEI to 2 kDa, a PEI‐melittin conjugate became an effective carrier of mRNA.^51^ Similar to PEI and other carriers, HK carriers differed in their ability to carry nucleic acids. Because HK carriers were made on a peptide synthesizer, their amino sequence can be varied and carefully controlled, and these differences in the sequences may affect the binding and release of the nucleic acids, as well as the stability of the polyplexes.^52^

In prior studies, the four‐branched HK peptide, H2K4b, was a good carrier of large molecular weight plasmids,^28^ although it was a poor carrier of the relatively low molecular weight siRNA.^40^ Previous data from our laboratory showed that the two histidine‐rich peptides, H3K4b and H3K(+H)4b, were effective carriers of siRNA,^40^, ^52^ although H3K(+H)4b appeared to be modestly more effective.^53^ Moreover, the H3K4b carrier of siRNA induced cytokines to a significantly greater degree *in vitro* and *in vivo* than the H3K(+H)4b‐siRNA polyplexes.^53^ In the present study, we determined that the H3K(+H)4b‐mRNA polyplex was approximately 10‐fold more efficient in expressing luciferase in MDA‐MB‐231 cells compared to H3K4b polyplex. Thus, the addition of a single histidine to the second domain of H3K4b enhanced mRNA transfection.

The addition of histidines to the branched HK did not necessarily improve the efficacy of the carrier in transporting mRNA. For example, the addition of two histidines to the N‐terminal ends of the branches of H3K(+H)4b (Table 1) did not increase luciferase expression. Nevertheless, all five of the branched HK carriers with the extra histidine in the second domain [H3K(+H)4b, H3k(+H)4b, H‐H3K(+H)4b, HH‐H3K(+H)4b, and H4K4b] were effective carriers of mRNA. Interestingly, these branched HK polyplexes demonstrated similar stability to various amounts of added heparin (see Supporting information, Figure S1). Moreover, the four‐branched HK peptides without an extra histidine in the second domain [H3K4b, H3K(1+H)4b, H3K(3+H)4b and H3K(1,3+H)4b] were not effective carriers of mRNA. These results suggest that an extra histidine in the second ‐HHHK motif of the branched of H3K(+H)4b significantly enhanced mRNA transfection efficiency.

At least part of the transfection differences between H3K(+H)4b and H3K4b particles appear to be a result of the structural and biophysical differences. As the gel retardation and heparin displacement assays demonstrated, the H3K(+H)4b polyplexes showed greater stability than H3K4b. Moreover, although these two HK polyplexes have a similar size when formed in water, the polyplexes of H3K4b were markedly larger when formed in Opti‐MEM or serum. The smaller and more stable particles formed by H3K(+H)4b could favor enhanced cellular uptake via endocytosis and contribute to enhanced intracellular mRNA delivery.

The enhanced stability of H3K(+H)4b polyplexes was further illustrated by the fluorescence images of the nanoparticles within the cell. The fluorescence of H3K(+H)4b‐mRNA polyplexes overlapped the acidic endosomal vesicles to a significant degree, whereas the fluorescence of H3K4b‐mRNA polyplexes overlapped to a much lesser degree. Moreover, the irregular‐shaped extracellular H3K4b polyplexes, which did not overlap with endosomes, were not observed with H3K(+H)4b polyplexes (Figure 5A) and the results suggest that decreased uptake may be a primary reason of the inefficiency of H3K4b carrier. In addition to the reduced uptake by H3K4b polyplexes, the increased release of mRNA from H3K4b polyplexes may play a role in the reduced transfection compared to H3K(+H)4b polyplexes. Together, these data complemented one another, indicating that the H3K4b polyplexes were less stable compared to H3K(+H)4b. We suggest that the additional histidine in the second domain enhanced transfection through increased stability of the polyplexes, perhaps by non‐ionic interactions between the polymers.

Analogous to other carries of nucleic acids including mRNA,^15^ selective pegylation and cross‐linking of HK carriers is expected to augment the stability of the polyplex *in vivo*. Nevertheless, the use of liposomal–polymer combinations or the polymer–lipid conjugates may be necessary for efficient mRNA transfection *in vivo* and, consequently, we initiated studies to investigate synergism between HK peptides and liposomes. The combination of DOTAP and H3K(+H)4b carriers was found to be synergistic with respect to their ability to carry mRNA into cells. In addition to DOTAP liposomes, we anticipate that several other cationic liposomes [i.e. 1,3‐dioleoyloxy‐2‐(6‐carboxy‐spermyl)‐propylamide] will be synergistic with the H3K(+H)4b polymers based on prior studies.^45^ Investigators have determined that incorporating helper lipids such as DOPE with DOTAP into liposomes may significantly enhance the cytotoxic T‐cell response.^54^ Furthermore, substituting an imidazole/histamine liposome for DOTAP liposomes, together with an histidylated polymer, markedly augmented the silencing activity of siRNA.^33^ Although this later study utilized siRNA, these differences between lipid preparations may extend to mRNA. Alternatively, we are also investigating whether HK lipopolymers will be effective carriers of mRNA. Conjugating fatty acyl chains or cholesterol to polymeric carriers of mRNA have markedly enhanced their ability to transfect cells and tissues *in vivo*.^6^, ^24^, ^55^

In summary, we have investigated a series of four‐branched HK peptides for mRNA delivery. Among them, we found that H3K(+H)4b was a significantly more effective carrier of mRNA than H3K4b. With the use of the heparin displacement assays, H3K(+H)4b‐mRNA polyplexes were determined to have greater stability than H3K4b polyplexes. Based on flow cytometry and fluorescent microscopy studies, the marked reduction in transfection by H3K4b polyplexes was likely a result of their decreased cellular uptake and accumulation within endosomes compared to the H3K(+H)4b polyplexes. Similar to the H3K(+H)4b peptides, several branched HK analogues with an extra histidine in the second motif enhanced mRNA transfection as well (Figure 6; see also Supporting information, Table S2). In addition to the extra histidine in the second domain of the branches, the other important factor in transfection with the HK peptides was the replacement of l‐lysines in the branches with d‐lysines. By its likely resistance to enzymatic degradation, the d‐analog of H3K(+H)4b [i.e. H3k(+H)4b] enhanced mRNA transfection by approximately 35% compared to the l‐analog with or without liposomes. Furthermore, significantly higher and synergistic levels of luciferase expression were observed with the combination of DOTAP and H3K(+H)4b [or H3k(+H)4b] than with either carrier alone. Based on these initial promising studies, more investigations are warranted for these mRNA transfection agents.

## AUTHOR CONTRIBUTIONS

AJM and JH designed the study and prepared the manuscript. JH, SX and AJM performed the heparin displacement and transfection studies. JH and QL carried out the uptake and fluorescent microscopy studies. JH conducted the size and zeta potential experiments. JH and AJM performed the statistical analysis.

## CONFLICT OF INTEREST STATEMENT

AJM has equity in Sirnaomics, Inc. A patent application has been filed on this work by the University of Maryland School of Medicine.

## Supporting information


**Figure S1.** Heparin displacement assays. After the polyplexes were formed (w:w; HK:mRNA; 4 μg:1 μg) and incubated with several concentrations of heparin, the Sybr Gold nucleic acid dye was incubated with the polyplexes for 5 minutes. Fluorescence was then measured by a microplate fluorimeter.
**Figure S2.** Uptake of cyanine 5‐labeled mRNA uptake into MDA‐MB‐231 cells with different peptide carriers. The percentages of cells containing labeled mRNA 1, 2 and 4 hours after transfection with H3K(+H)4b (upper) or with H3K4b (lower).
**Figure S3.** Stability of HK polyplexes to enzymatic degradation. After preparation of the H3K4b or H3k(+H)4b mRNA polyplexes [w:w; HK (0.5, 1 and 4 μg):mRNA (1 μg)], these polyplexes were incubated with trypsin (0.025%) for 30 or 60 minutes. The HK polyplexes were then loaded on a 1% agarose gel and electrophoresis was carried out at 75 V for 30 minutes in TAE buffer. The gel was stained in a TAE buffer containing ethidium bromide (1 μg/ml) for 10 minutes. As evidenced by the release of mRNA from the polyplex, the H3K(+H)4b mRNA polyplex showed reduced stability to trypsin at ratios of 1:2 and 1:1 compared to the H3k(+H)4b polyplex.
**Figure S4.** Transfection of mRNA (1 μg) with HK peptides (4 μg) and/or DOTAP liposomes (1 μg). Cells were transfected with mRNA lipoplexes, polyplexes, or lipopolyplexes as described in the Materials and methods, and 24 hours later, luciferase activity was measured. ***p < 0.001; ****p < 0.0001
**Table S1.** Trypan blue exclusion method
**Table S2.** Transfection of mRNA with four‐branched HK peptides

## Data Availability

The data that support the findings of this study are available from the corresponding author upon reasonable request.

## References

[1] Zohra FT , Maitani Y , Akaike T . mRNA delivery through fibronectin associated liposome‐apatite particles: a new approach for enhanced mRNA transfection to mammalian cell. Biol Pharm Bull. 2012;35:111‐115. 22223346 10.1248/bpb.35.111

[2] Phua KK , Leong KW , Nair SK . Transfection efficiency and transgene expression kinetics of mRNA delivered in naked and nanoparticle format. J Control Release. 2013;166:227‐233. 23306021 10.1016/j.jconrel.2012.12.029 PMC3594075

[3] Pardi N , Tuyishime S , Muramatsu H , et al. Expression kinetics of nucleoside‐modified mRNA delivered in lipid nanoparticles to mice by various routes. J Control Release. 2015;217:345‐351. 26264835 10.1016/j.jconrel.2015.08.007 PMC4624045

[4] Kowalski PS , Rudra A , Miao L , Anderson DG . Delivering the messenger: advances in technologies for therapeutic mRNA delivery. Mol Ther. 2019;27:710‐728. 30846391 10.1016/j.ymthe.2019.02.012 PMC6453548

[5] Freitag F , Wagner E . Optimizing synthetic nucleic acid and protein nanocarriers: the chemical evolution approach. Adv Drug Deliv Rev. 2020 10.1016/j.addr.2020.03.005 32246984

[6] Jarzebinska A , Pasewald T , Lambrecht J , et al. A single methylene group in oligoalkylamine‐based cationic polymers and lipids promotes enhanced mRNA delivery. Angew Chem Int Ed Engl. 2016;55:9591‐9595. 27376704 10.1002/anie.201603648

[7] McKinlay CJ , Benner NL , Haabeth OA , Waymouth RM , Wender PA . Enhanced mRNA delivery into lymphocytes enabled by lipid‐varied libraries of charge‐altering releasable transporters. Proc Natl Acad Sci U S A. 2018;115:E5859‐E5866. 29891683 10.1073/pnas.1805358115 PMC6042134

[8] McKinlay CJ , Vargas JR , Blake TR , et al. Charge‐altering releasable transporters (CARTs) for the delivery and release of mRNA in living animals. Proc Natl Acad Sci U S A. 2017;114:E448‐E456. 28069945 10.1073/pnas.1614193114 PMC5278438

[9] Wang F , Xiao W , Elbahnasawy MA , et al. Optimization of the linker length of mannose‐cholesterol conjugates for enhanced mRNA delivery to dendritic cells by liposomes. Front Pharmacol. 2018;9:980 10.3389/fphar.2018.00980 30233368 PMC6134263

[10] Badieyan ZS , Pasewald T , Mykhaylyk O , Rudolph C , Plank C . Efficient ex vivo delivery of chemically modified messenger RNA using lipofection and magnetofection. Biochem Biophys Res Commun. 2017;482:796‐801. 27888105 10.1016/j.bbrc.2016.11.113

[11] Zohra FT , Chowdhury EH , Akaike T . High performance mRNA transfection through carbonate apatite‐cationic liposome conjugates. Biomaterials. 2009;30:4006‐4013. 19410288 10.1016/j.biomaterials.2009.02.050

[12] Uchida H , Itaka K , Nomoto T , et al. Modulated protonation of side chain aminoethylene repeats in N‐substituted polyaspartamides promotes mRNA transfection. J Am Chem Soc. 2014;136:12396‐12405. 25133991 10.1021/ja506194z

[13] Crowley ST , Poliskey JA , Baumhover NJ , Rice KG . Efficient expression of stabilized mRNA PEG‐peptide polyplexes in liver. Gene Ther. 2015;22:993‐999. 26125604 10.1038/gt.2015.68 PMC4670273

[14] Nuhn L , Kaps L , Diken M , Schuppan D , Zentel R . Reductive Decationizable block copolymers for stimuli‐responsive mRNA delivery. Macromol Rapid Commun. 2016;37:924‐933. 27075781 10.1002/marc.201600046

[15] Chen Q , Qi R , Chen X , et al. A targeted and stable polymeric nanoformulation enhances systemic delivery of mRNA to tumors. Mol Ther. 2017;25:92‐101. 28129133 10.1016/j.ymthe.2016.10.006 PMC5363296

[16] Li J , He Y , Wang W , Wu C , Hong C , Hammond PT . Polyamine‐mediated stoichiometric assembly of ribonucleoproteins for enhanced mRNA delivery. Angew Chem Int Ed Engl. 2017;56:13709‐13712. 28925033 10.1002/anie.201707466 PMC5647255

[17] Udhayakumar VK , De Beuckelaer A , McCaffrey J , et al. Arginine‐rich peptide‐based mRNA nanocomplexes efficiently instigate cytotoxic T cell immunity dependent on the amphipathic organization of the peptide. Adv Healthc Mater. 2017;6 10.1002/adhm.201601412 28436620

[18] Capasso Palmiero U , Kaczmarek JC , Fenton OS , Anderson DG . Poly (beta‐amino ester)‐co‐poly (caprolactone) terpolymers as nonviral vectors for mRNA delivery in vitro and in vivo. Adv Healthc Mater. 2018;7:e1800249 10.1002/adhm.201800249 29761648 PMC13574336

[19] Kowalski PS , Capasso Palmiero U , Huang Y , Rudra A , Langer R , Anderson DG . Ionizable amino‐polyesters synthesized via ring opening polymerization of tertiary amino‐alcohols for tissue selective mRNA delivery. Adv Mater. 2018;30:e1801151 10.1002/adma.201801151 PMC6320729 29975801

[20] Kaczmarek JC , Kauffman KJ , Fenton OS , et al. Optimization of a degradable polymer‐lipid nanoparticle for potent systemic delivery of mRNA to the lung endothelium and immune cells. Nano Lett. 2018;18:6449‐6454. 30211557 10.1021/acs.nanolett.8b02917 PMC6415675

[21] Qiu Y , Man RCH , Liao Q , Kung KLK , Chow MYT , Lam JKW . Effective mRNA pulmonary delivery by dry powder formulation of PEGylated synthetic KL4 peptide. J Control Release. 2019;314:102‐115. 31629037 10.1016/j.jconrel.2019.10.026

[22] Chan LY , Khung YL , Lin CY . Preparation of messenger RNA nanomicelles via non‐cytotoxic PEG‐polyamine nanocomplex for intracerebroventicular delivery: a proof‐of‐concept study in mouse models. Nanomaterials (Basel). 2019;9:67 10.3390/nano9010067 PMC6359661 30621291

[23] Patel AK , Kaczmarek JC , Bose S , et al. Inhaled nanoformulated mRNA polyplexes for protein production in lung epithelium. Adv Mater. 2019;31:e1805116 10.1002/adma.201805116 30609147 PMC7490222

[24] Uchida S , Kinoh H , Ishii T , et al. Systemic delivery of messenger RNA for the treatment of pancreatic cancer using polyplex nanomicelles with a cholesterol moiety. Biomaterials. 2016;82:221‐228. 26763736 10.1016/j.biomaterials.2015.12.031

[25] Chen G , Ma B , Wang Y , Gong S . A universal GSH‐responsive nanoplatform for the delivery of DNA, mRNA, and Cas9/sgRNA ribonucleoprotein. ACS Appl Mater Interfaces. 2018;10:18515‐18523. 29798662 10.1021/acsami.8b03496 PMC6141193

[26] Salmasi Z , Shier WT , Hashemi M , et al. Heterocyclic amine‐modified polyethylenimine as gene carriers for transfection of mammalian cells. Eur J Pharm Biopharm. 2015;96:76‐88. 26209125 10.1016/j.ejpb.2015.07.008

[27] Lachelt U , Kos P , Mickler FM , et al. Fine‐tuning of proton sponges by precise diaminoethanes and histidines in pDNA polyplexes. Nanomedicine. 2014;10:35‐44. 10.1016/j.nano.2013.07.008 23891984

[28] Leng Q , Mixson AJ . Modified branched peptides with a histidine‐rich tail enhance in vitro gene transfection. Nucleic Acids Res. 2005;33:e40 10.1093/nar/gni040 15731333 PMC549579

[29] Wang F , Wang Y , Wang H , Shao N , Chen Y , Cheng Y . Synergistic effect of amino acids modified on dendrimer surface in gene delivery. Biomaterials. 2014;35:9187‐9198. 25112938 10.1016/j.biomaterials.2014.07.027

[30] Chang KL , Higuchi Y , Kawakami S , Yamashita F , Hashida M . Efficient gene transfection by histidine‐modified chitosan through enhancement of endosomal escape. Bioconjug Chem. 2010;21:1087‐1095. 20499901 10.1021/bc1000609

[31] Chang KL , Higuchi Y , Kawakami S , Yamashita F , Hashida M . Development of lysine‐histidine dendron modified chitosan for improving transfection efficiency in HEK293 cells. J Control Release. 2011;156:195‐202. 21802461 10.1016/j.jconrel.2011.07.021

[32] He J , Xu S , Mixson AJ . The multifaceted histidine‐based carriers for nucleic acid delivery: advances and challenges. Pharmaceutics. 2020;12 10.3390/pharmaceutics12080774 PMC7465012 32823960

[33] Goncalves C , Berchel M , Gosselin MP , et al. Lipopolyplexes comprising imidazole/imidazolium lipophosphoramidate, histidinylated polyethyleneimine and siRNA as efficient formulation for siRNA transfection. Int J Pharm. 2014;460:264‐272. 24225347 10.1016/j.ijpharm.2013.11.005

[34] Midoux P , Pichon C , Yaouanc JJ , Jaffrès PA . Chemical vectors for gene delivery: a current review on polymers, peptides and lipids containing histidine or imidazole as nucleic acids carriers. Br J Pharmacol. 2009;157:166‐178. 19459843 10.1111/j.1476-5381.2009.00288.x PMC2697805

[35] Mockey M , Bourseau E , Chandrashekhar V , et al. mRNA‐based cancer vaccine: prevention of B16 melanoma progression and metastasis by systemic injection of MART1 mRNA histidylated lipopolyplexes. Cancer Gene Ther. 2007;14:802‐814. 17589432 10.1038/sj.cgt.7701072

[36] Perche F , Benvegnu T , Berchel M , et al. Enhancement of dendritic cells transfection in vivo and of vaccination against B16F10 melanoma with mannosylated histidylated lipopolyplexes loaded with tumor antigen messenger RNA. Nanomedicine. 2011;7:445‐453. 21220051 10.1016/j.nano.2010.12.010

[37] Gao H , Goncalves C , Gallego T , et al. Comparative binding and uptake of liposomes decorated with mannose oligosaccharides by cells expressing the mannose receptor or DC‐SIGN. Carbohydr Res. 2020;487:107877 10.1016/j.carres.2019.107877 31766009

[38] Le Moignic A , Malard V , Benvegnu T , et al. Preclinical evaluation of mRNA trimannosylated lipopolyplexes as therapeutic cancer vaccines targeting dendritic cells. J Control Release. 2018;278:110‐121. 29630987 10.1016/j.jconrel.2018.03.035

[39] Zhang L , Ambulos N , Mixson AJ . DNA delivery to cells in culture using peptides. Methods Mol Biol. 2004;245:33‐52. 14707368 10.1385/1-59259-649-5:33

[40] Leng Q , Scaria P , Zhu J , Ambulos N , Campbell P , Mixson AJ . Highly branched HK peptides are effective carriers of siRNA. J Gene Med. 2005;7:977‐986. 15772938 10.1002/jgm.748

[41] Strober W . Trypan blue exclusion test of cell viability. Curr Protoc Immunol. 2015;111:A3.B.1–A3.B.3.1. 10.1002/0471142735.ima03bs111 PMC6716531 26529666

[42] Schindelin J , Arganda‐Carreras I , Frise E , et al. Fiji: an open‐source platform for biological‐image analysis. Nat Methods. 2012;9:676‐682. 22743772 10.1038/nmeth.2019 PMC3855844

[43] Chou ST , Leng Q , Scaria P , Woodle M , Mixson AJ . Selective modification of HK peptides enhances siRNA silencing of tumor targets in vivo. Cancer Gene Ther. 2011;18:707‐716. 21818135 10.1038/cgt.2011.40 PMC3177007

[44] Leng Q , Woodle MC , Liu Y , Mixson AJ . Silver adducts of four‐branched histidine rich peptides exhibit synergistic antifungal activity. Biochem Biophys Res Commun. 2016;477:957‐962. 27387239 10.1016/j.bbrc.2016.07.008 PMC4968200

[45] Chen QR , Zhang L , Stass SA , Mixson AJ . Co‐polymer of histidine and lysine markedly enhances transfection efficiency of liposomes. Gene Ther. 2000;7:1698‐1705. 11083479 10.1038/sj.gt.3301294

[46] Blakney AK , Yilmaz G , McKay PF , Becer CR , Shattock RJ . One size does not fit all: the effect of chain length and charge density of poly (ethylene imine) based copolymers on delivery of pDNA, mRNA, and RepRNA polyplexes. Biomacromolecules. 2018;19:2870‐2879. 29698602 10.1021/acs.biomac.8b00429

[47] Goncalves C , Akhter S , Pichon C , et al. Intracellular availability of pDNA and mRNA after transfection: a comparative study among polyplexes, lipoplexes, and lipopolyplexes. Mol Pharm. 2016;13:3153‐3163. 27486998 10.1021/acs.molpharmaceut.6b00376

[48] Kauffman AC , Piotrowski‐Daspit AS , Nakazawa KH , Jiang Y , Datye A , Saltzman WM . Tunability of biodegradable poly(amine‐co‐ester) polymers for customized nucleic acid delivery and other biomedical applications. Biomacromolecules. 2018;19:3861‐3873. 30110158 10.1021/acs.biomac.8b00997 PMC6510397

[49] Peng L , Wagner E . Polymeric carriers for nucleic acid delivery: current designs and future directions. Biomacromolecules. 2019;20:3613‐3626. 31497946 10.1021/acs.biomac.9b00999

[50] Scholz C , Wagner E . Therapeutic plasmid DNA versus siRNA delivery: common and different tasks for synthetic carriers. J Control Release. 2012;161:554‐565. 22123560 10.1016/j.jconrel.2011.11.014

[51] Bettinger T , Carlisle RC , Read ML , Ogris M , Seymour LW . Peptide‐mediated RNA delivery: a novel approach for enhanced transfection of primary and post‐mitotic cells. Nucleic Acids Res. 2001;29:3882‐3891. 11557821 10.1093/nar/29.18.3882 PMC55922

[52] Chou ST , Hom K , Zhang D , et al. Enhanced silencing and stabilization of siRNA polyplexes by histidine‐mediated hydrogen bonds. Biomaterials. 2014;35:846‐855. 24161165 10.1016/j.biomaterials.2013.10.019 PMC3920840

[53] Leng Q , Chou ST , Scaria PV , Woodle MC , Mixson AJ . Buffering capacity and size of siRNA polyplexes influence cytokine levels. Mol Ther. 2012;20:2282‐2290. 23032972 10.1038/mt.2012.206 PMC3519993

[54] Hess PR , Boczkowski D , Nair SK , Snyder D , Gilboa E . Vaccination with mRNAs encoding tumor‐associated antigens and granulocyte‐macrophage colony‐stimulating factor efficiently primes CTL responses, but is insufficient to overcome tolerance to a model tumor/self antigen. Cancer Immunol Immunother. 2006;55:672‐683. 16133108 10.1007/s00262-005-0064-z PMC11030883

[55] Kaczmarek JC , Patel AK , Kauffman KJ , et al. Polymer‐lipid nanoparticles for systemic delivery of mRNA to the lungs. Angew Chem Int Ed Engl. 2016;55:13808‐13812. 27690187 10.1002/anie.201608450 PMC5279893

