# Supplementary material for: Location of a single histidine within peptide carriers increases mRNA delivery
Source: J Gene Med. 2020 Dec 21;23(2):e3295. doi: 10.1002/jgm.3295 (PMC7900953; doi:10.1002/jgm.3295)
Supplement: Supplementary file 1 — Figure S1. Heparin displacement assays. After the polyplexes were formed (w:w; HK:mRNA; 4 μg:1 μg) and incubated with several concentrations of heparin, the Sybr Gold nucleic acid dye was incubated with the polyplexes for 5 minutes. Fluorescence was then measured by a microplate fluorimeter. Figure S2. Uptake of cyanine 5‐labeled mRNA uptake into MDA‐MB‐231 cells with different peptide carriers. The percentages of cells containing labeled mRNA 1, 2 and 4 hours after transfection with H3K(+H)4b (upper) or with H3K4b (lower). Figure S3. Stability of HK polyplexes to enzymatic degradation. After preparation of the H3K4b or H3k(+H)4b mRNA polyplexes [w:w; HK (0.5, 1 and 4 μg):mRNA (1 μg)], these polyplexes were incubated with trypsin (0.025%) for 30 or 60 minutes. The HK polyplexes were then loaded on a 1% agarose gel and electrophoresis was carried out at 75 V for 30 minutes in TAE buffer. The gel was stained in a TAE buffer containing ethidium bromide (1 μg/ml) for 10 minutes. As evidenced by the release of mRNA from the polyplex, the H3K(+H)4b mRNA polyplex showed reduced stability to trypsin at ratios of 1:2 and 1:1 compared to the H3k(+H)4b polyplex. Figure S4. Transfection of mRNA (1 μg) with HK peptides (4 μg) and/or DOTAP liposomes (1 μg). Cells were transfected with mRNA lipoplexes, polyplexes, or lipopolyplexes as described in the Materials and methods, and 24 hours later, luciferase activity was measured. ***p < 0.001; ****p < 0.0001 Table S1. Trypan blue exclusion method Table S2. Transfection of mRNA with four‐branched HK peptides [file JGM-23-e3295-s001.docx]

**Supporting Information**

**Location of a single histidine within peptide carriers increased mRNA delivery**

Jiaxi He, Songhui Xu, Qixin Leng, and A. James Mixson


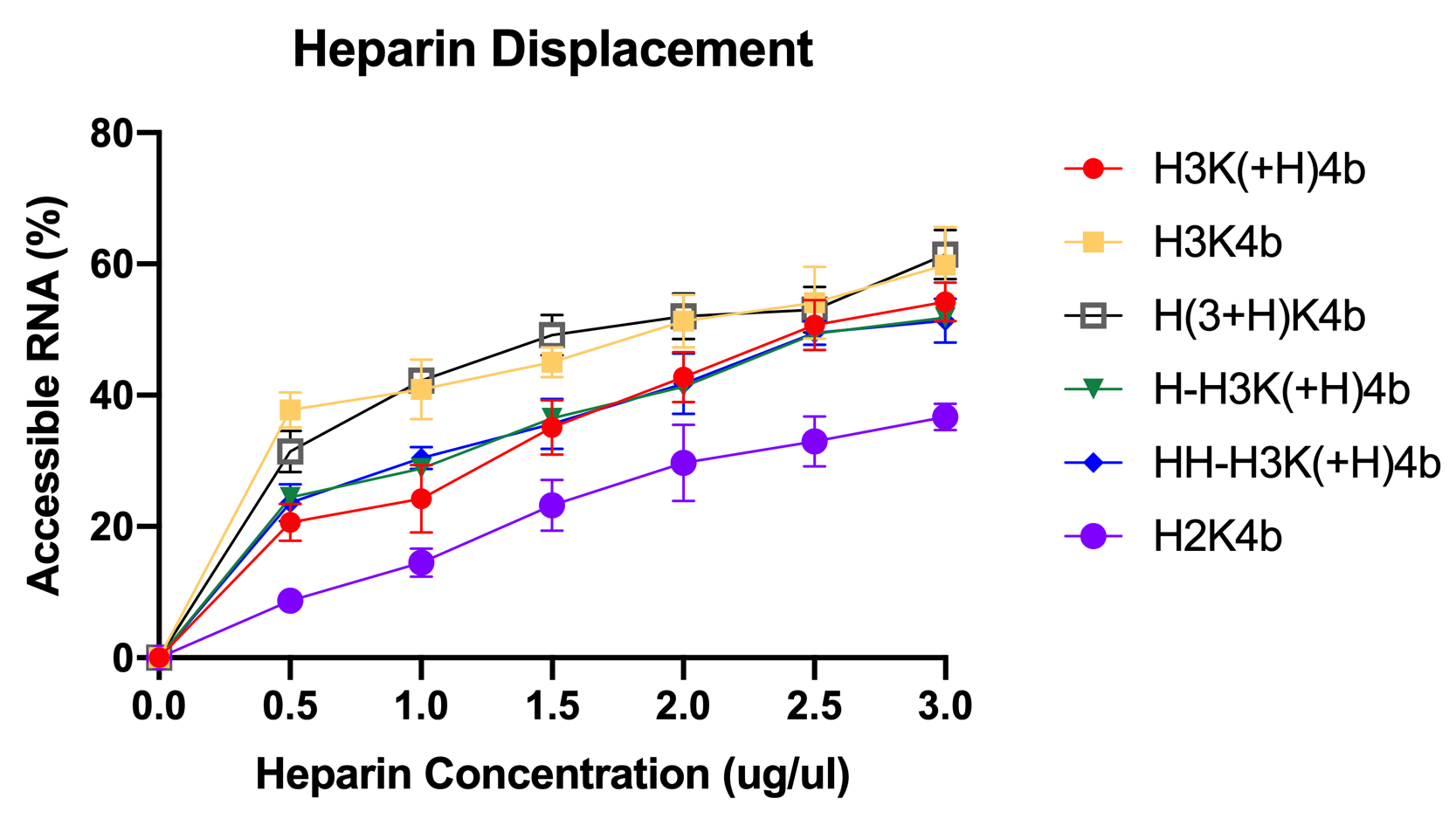


**Figure S1.** Heparin Displacement Assays. After the polyplexes were formed (wt:wt; HK:mRNA; 4 μg:1 μg) and incubated with several concentrations of heparin, the Sybr Gold nucleic acid dye was incubated with the polyplexes for 5 mins. Fluorescence was then measured by a microplate fluorimeter.


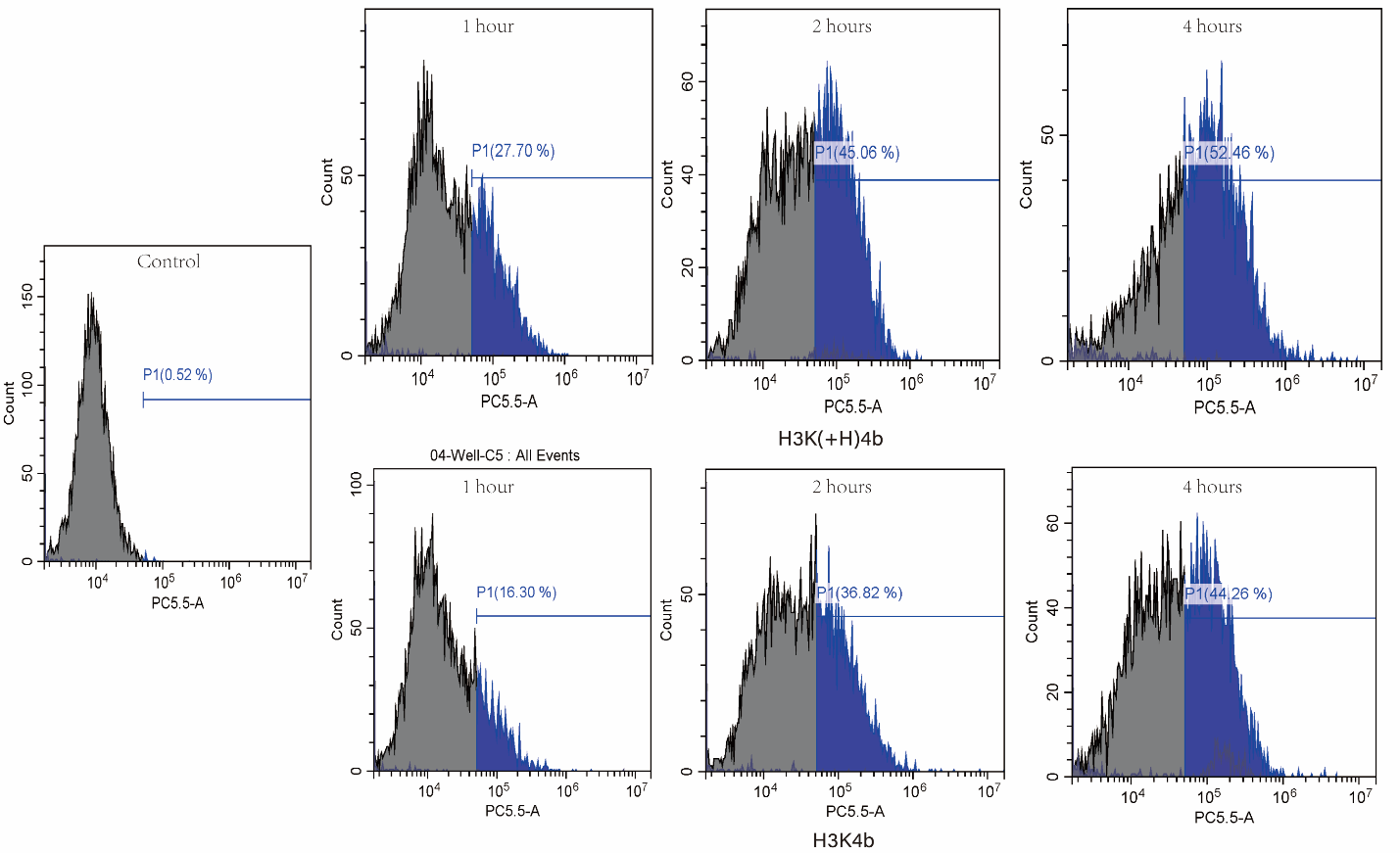


**Figure S2.** Uptake of cyanine 5-labeled mRNA uptake into MDA-MB-231 cells with different peptide carriers. The percentages of cells containing labeled mRNA 1, 2, 4 hours after transfection with H3K(+H)4b (upper panel) or with H3K4b (lower panel).

**
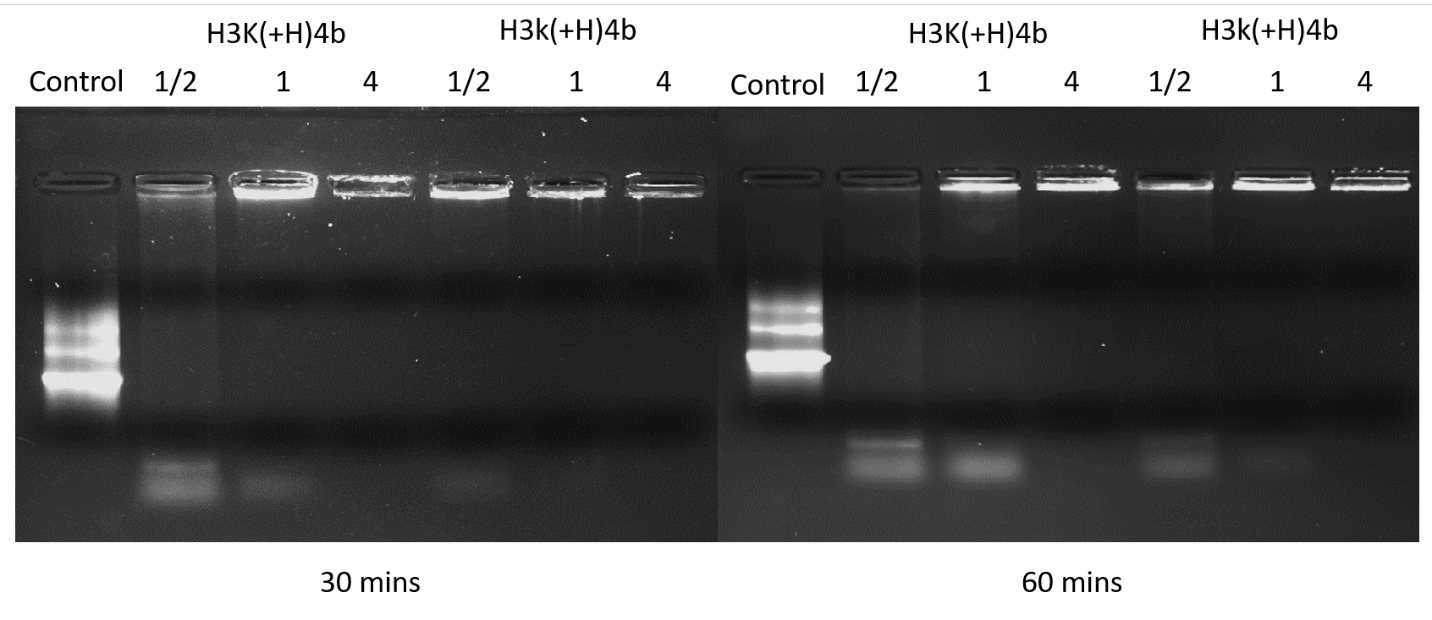
Figure S3.** Stability of HK polyplexes to enzymatic degradation. After preparation of the H3K4b or H3k(+H)4b mRNA polyplexes (wt:wt; HK (0.5, 1, 4 μg):mRNA (1 μg)), these polyplexes were incubated with trypsin (0.025%) for 30 or 60 min. The HK polyplexes were then loaded on a 1% agarose gel and electrophoresis was carried out at 75 V for 30 min in TAE buffer. The gel was stained in a TAE buffer containing ethidium bromide (1 μg/ml) for 10 min. As evidenced by the release of mRNA from the polyplex, the H3K(+H)4b mRNA polyplex showed reduced stability to trypsin at the 1:2 and 1:1 ratios compared to the H3k(+H)4b polyplex.

**
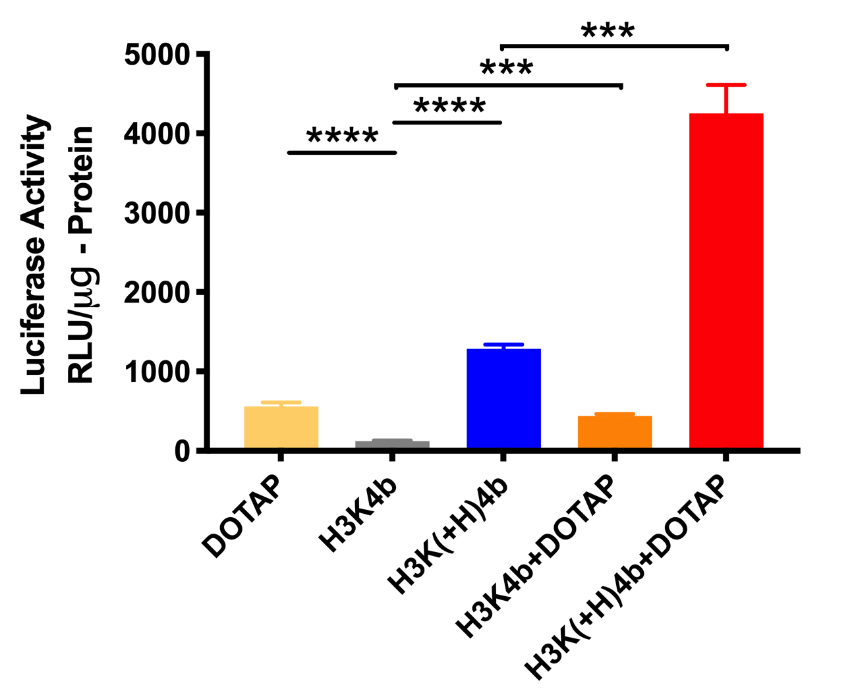
**

**Figure S4.** Transfection of mRNA (1 μg) with HK peptides (4 μg) and/or DOTAP liposomes (1 μg). Cells were transfected with mRNA lipoplexes, polyplexes, or lipopolyplexes as described in the Material and Methods section, and twenty-four h later, the luciferase activity was measured. ***, P<0.001; ****, P<0.0001

**Table S1. Trypan Blue Exclusion Method**

| **Treatment** | **% Viability** |
| --- | --- |
| Untreated Cells | 97.6 |
| H3K4b | 95.6 |
| H3K4b + mRNA | 95.4 |
| H3(+H)K4b | 96.3 |
| H3(+H)K4b + mRNA | 94.3 |

After the medium was changed to Opti-MEM, either the polymer (4 μg) or the polyplex (4 μg HK; 1 μg mRNA) was added to the cells for 5 h. The media was then changed to DMEM/10% FBS for 19 h and the cell viability was determined using the trypan cell exclusion assay.

**Table S2. Transfection of mRNA with four-branched HK Peptides**

| **Polymers** | **Ratio(wt:wt;mRNA:Polymer)** | **RLU/μg-Protein** |
| --- | --- | --- |
|  |  |  |
| H3K(+H)4b | 1:4 | 1532.9±122.9 |
|  | 1:8 | 1656.3±202.5 |
|  | 1:12 | 1033.4±197 |
|  | 1:4 | 1851.6±138.3 |
| H3k(+H)4b | 1:8 | 1787.2±195.2 |
|  | 1:12 | 1982.3±210.7 |
| H3K4b | 1:4 | 156.8±41.8 |
|  | 1:8 | 62.1±13.2 |
|  | 1:12 | 18.1±4.0 |
| H3K(3+H)4b | 1:4 | 61.7±5.7 |
|  | 1:8 | 68.7±3.1 |
|  | 1:12 | 59.0±7.5 |
| H3K(1+H)4b | 1:4 | 24.3±4.5 |
|  | 1:8 | 15.0±3.6 |
|  | 1:12 | 7.3±2.5 |
| H-H3K(+H)4b | 1:4 | 1107.5±140.4 |
|  | 1:8 | 874.6±65.2 |
|  | 1:12 | 676.4±25.7 |
| HH-H3K(+H)4b | 1:4 | 1101.9±106.6 |
|  | 1:8 | 832.2±75.3 |
|  | 1:12 | 739.8±105.4 |
| H4K4b | 1:4 | 896.4±112.6 |
|  | 1:8 | 821.8±115.6 |
|  | 1:12 | 522.4±69.2 |
| H3(1,3+H)K4b | 1:4 | 518.3±134.7 |
|  | 1:8 | 427.7±18.1 |
|  | 1:12 | 378±5.2 |
|  | 1:4 | 546.7±70.1 |
| H2K4b | 1:8 | 132.3±58.5 |
|  | 1:12 | 194.7±18.4 |
| Negative Control | - | 0.0153±0.006 |

To prepare HK polyplexes, luciferase-expressing mRNA (1 μg) in 50 μl of Opti-MEM was briefly mixed with one of the HK peptides (4, 8, or 12 μg) and maintained at room temperature for 30 min. This polyplex was then added dropwise to the MDA-MB-231 cells and after four h, the Opti-MEM media was removed and replaced with DMEM/10% serum. Twenty-four hours later, luciferase activity was measured.
